# A combined NMR and EPR investigation on the effect of the disordered RGG regions in the structure and the activity of the RRM domain of FUS

**DOI:** 10.1038/s41598-020-77899-x

**Published:** 2020-12-01

**Authors:** A. Bonucci, M. G. Murrali, L. Banci, R. Pierattelli

**Affiliations:** 1 CERM - Magnetic Resonance Center, University of Florence, Via Luigi Sacconi 6, 50019 Sesto Fiorentino, Italy; 2 Department of Chemistry, University of Florence, Via della Lastruccia 3, 50019 Sesto Fiorentino, Italy

**Keywords:** Biophysics, Intrinsically disordered proteins, Molecular biophysics

## Abstract

Structural disorder represents a key feature in the mechanism of action of RNA-binding proteins (RBPs). Recent insights revealed that intrinsically disordered regions (IDRs) linking globular domains modulate their capability to interact with various sequences of RNA, but also regulate aggregation processes, stress-granules formation, and binding to other proteins. The FET protein family, which includes FUS (Fused in Sarcoma), EWG (Ewing Sarcoma) and TAF15 (TATA binding association factor 15) proteins, is a group of RBPs containing three different long IDRs characterized by the presence of RGG motifs. In this study, we present the characterization of a fragment of FUS comprising two RGG regions flanking the RNA Recognition Motif (RRM) alone and in the presence of a stem-loop RNA. From a combination of EPR and NMR spectroscopies, we established that the two RGG regions transiently interact with the RRM itself. These interactions may play a role in the recognition of stem-loop RNA, without a disorder-to-order transition but retaining high dynamics.

## Introduction

The “classic” RNA-binding proteins (RBPs) are a group of proteins playing central roles in the formation of ribonucleoprotein complexes as they participate in various gene expression processes. The capability of RBPs to bind RNA has been generally ascribed to the globular domains that possess a defined secondary structure, such as the RNA recognition motif (RRM), the K homology domain (KH), the zinc fingers (ZnF) and the DEAD box helicase motifs^1–4^.

Some studies revealed that 3–11% of the human proteome is involved in RNA binding, but only a small fraction of proteins could be classified as typical globular RBPs^5^. Indeed, recent reports revealed that intrinsically disordered regions (IDRs) are largely distributed in various RBPs sequences and contribute to RNA binding, cooperate with globular domains, and should play relevant roles in RNA interaction^6–9^.

The second most common class of intrinsically disordered regions in RBPs is represented by RGG-boxes (or GAR) that contain repeats of arginine and glycine residues. The absence of a folded conformation of the RGG motifs is due to the abundance of hydrophilic and charged amino acids and to the lack of bulky hydrophobic groups that promote the formation of stable three-dimensional structures. RGG repeats are usually associated with other repetitive sequences, including polyalanine, polyaspartate, polyglutamate, polyglutamine, and polyglycine repeats, as well as other diglycine-containing motifs such as FGG, PGG, SGG and YGG. The positively charged guanidinium group of arginine can form different types of interactions with different elements of nucleic acids, including cation-π interactions, π–π stacking, hydrogen bonding, charge-charge/dipole and van der Waals interactions. On the other hand, glycine is the smallest amino acid with a single hydrogen atom as side chain and therefore it represents the key-factor for the conformational flexibility of RGG domains. Moreover, post-translational modifications (PTM), such as arginine methylation, may represent another important factor for modulating the functions of the RGG boxes. PTMs could alter protein–protein and protein-RNA binding capability and could regulate the interaction of the protein with a group of RNA sequences and structures^6,10–12^.

**Figure 1:**
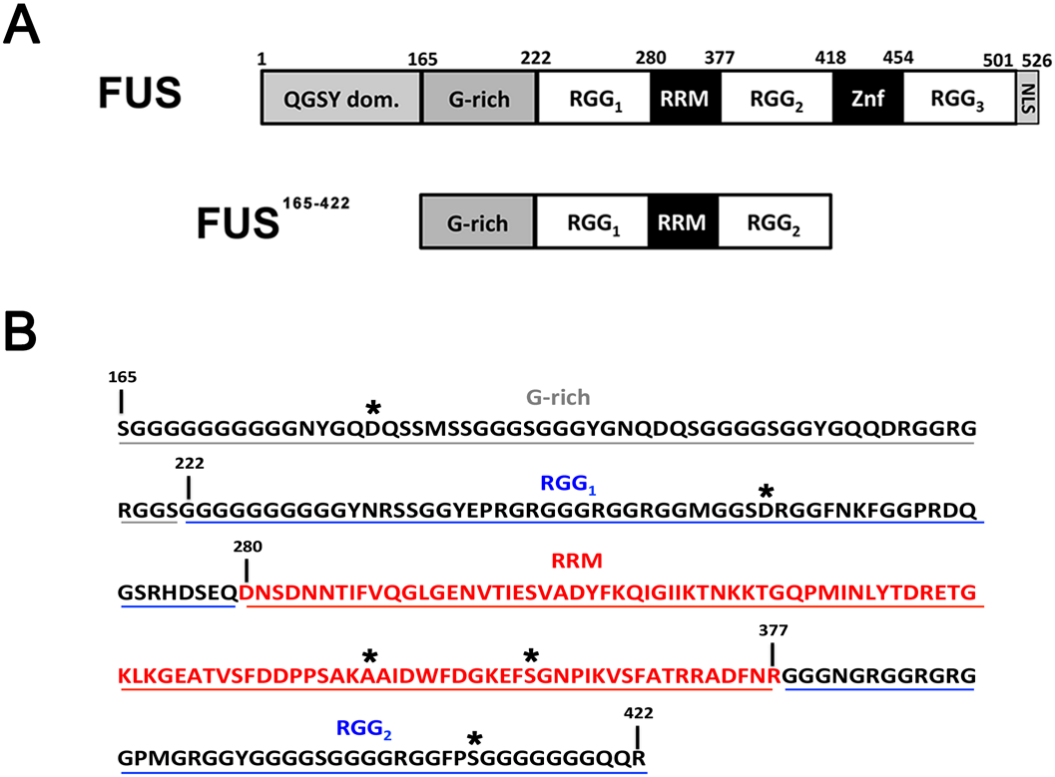
(**A**) Organization of full-length FUS and FUS^165–422^; (**B**) primary sequence of FUS^165–422^. The residues selected for replacement with cysteine and MTSL-labelling are marked with asterisk.

Fused in Sarcoma (FUS) protein is a nuclear protein implicated in the transcription, splicing and transport of mRNA. The scientific interest for this protein is due to its direct implication in neurodegenerative disorders, specifically in Amyotrophic Lateral Sclerosis (ALS)^13,14^. FUS is a multi-domain protein formed by a QGSY-rich region at the N-terminus, a globular RNA-recognition motif (RRM) with the classical βαββαβ fold, a zinc finger domain (ZnF), three different RGG boxes (RGG_1_, RGG_2_ and RGG_3_), a G-rich region and a C-terminal nuclear localization signal (NLS) (Fig. 1). RRM and ZnF represent the principal sites for RNA interaction, even if recent studies suggested a relevant role exerted by the RGG motifs in the RNA interaction and other cellular processes, such as phase-separation and stress granules formation^15–18^. In addition, the RGG boxes promote the affinity of folded domains for RNA possibly without taking a defined conformation during nucleotides binding. These disordered regions confer also the “degenerate-specificity” character in RNA interaction that is the capability of FUS to bind different nucleotides sequences and structures of RNA^18^. Moreover, the RGG_3_ and NLS regions are directly involved in the interaction with the nuclear import receptor Transportin/Karyopherin-β2, which translocates FUS into the nucleoplasm^16–21^.

In the present study we focused on the core RRM structured domain of FUS and on its two flanking IDRs both containing RGG motifs, to further characterize these parts of the protein and assess whether the interplay between the RGG_1-2_ and the RRM could be of importance in determining the function of the protein, particularly in the contest of RNA binding. Through a combined approach based on NMR and EPR spectroscopies, we provided insights concerning the conformation and dynamics of these domains, but also some hints on the possible roles exerted by RGGs regions in the RRM-RNA binding.

## Results

### Characterization of FUS^165–422^

Various studies highlighted the importance of the RGG domains in the physiological RNA-binding mechanism of the RRM of FUS^17,18^; however, biophysical investigations on a FUS fragment comprising the RRM and the two extended flanking RGG disordered regions (FUS^165–422^, Fig. 1) are very scarce. For this reason, we designed a plasmid encoding this fragment into pDEST-HisMBP vector and we expressed and purified the system using recombinant methodologies (see “Methods”)*.*

The resulting 258-residues long construct, with a molecular weight of 25 kDa, is composed of about 20% charged residues, 10% polar residues, and 37% glycine residues, with many repeats beside the canonical GG and RGG motives (i.e. SS, QQ, etc.). This unique content of glycine in FUS^165–422^ primary structure represents the key reason of the intrinsically disordered behavior of the RGG domains. Moreover, the high pI values of both unfolded domains (10.2 and 12.4 for RGG_1_ and RGG_2_, respectively) underline that these regions of FUS are positively charged under physiological conditions (pH 7.0–7.4), thus being capable of interacting with negative charges of nucleotides or others proteins. The main type of interactions between RBPs and RNA are π-π contacts, exerted by nucleobases and aromatic amino acids. Trp, Tyr and Phe residues in FUS^165–422^ are present in the RRM domain only, consistent with this region being the principal site for RNA interaction^22^.

**Figure 2:**
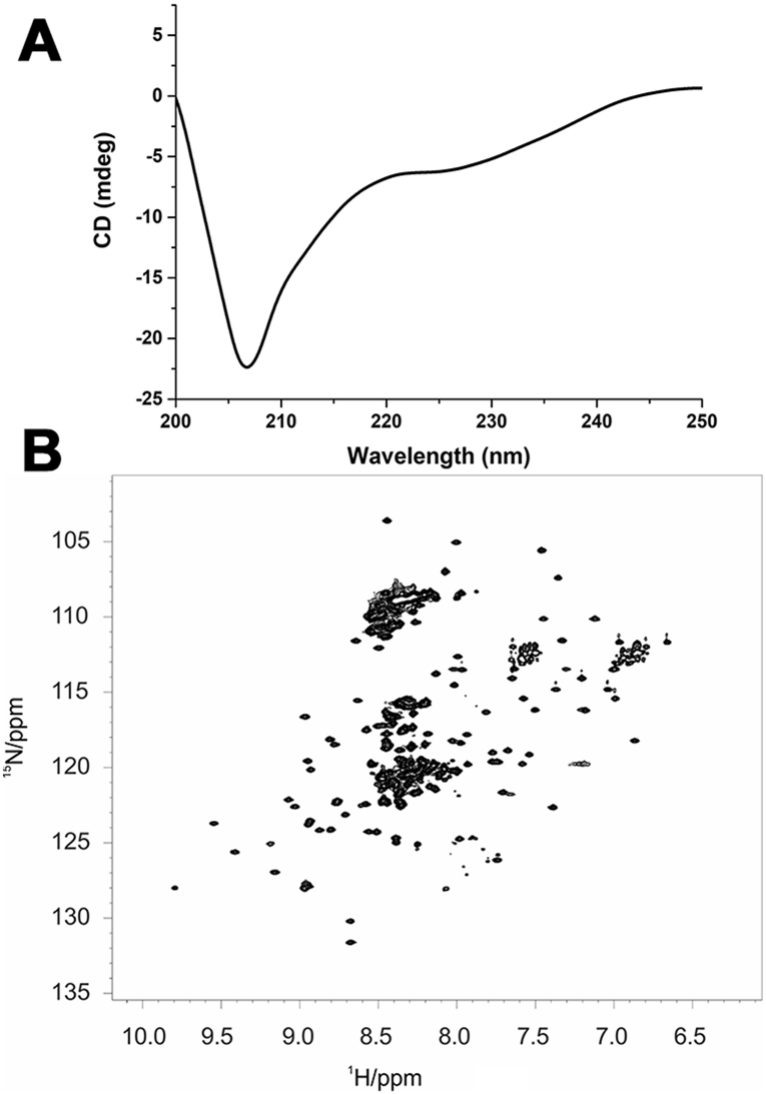
(**A**) Far-UV CD spectrum (200–250 nm) of FUS^165–422^; (**B**) 2D ^1^H-^15^N HSQC spectra of FUS^165–422^. The NMR spectrum was acquired at 298 K with a 22.3 T Bruker Avance III spectrometer equipped with a TCI probehead.

CD spectroscopy was used to confirm the secondary structure content present in FUS^165–422^. The far-UV CD spectrum of the protein in MES buffer solution at pH 6.5 (Fig. 2A) presents two peaks at 207 nm and 225 nm, a hallmark of the presence of helical conformation in the polypeptide, but the overall spectrum indicated the net presence of random coil structures as well. The percentage of secondary structural elements, estimated with the program Dichroweb^23^, accounts for 25% ± 3.1% of folded conformations (16% ± 3.1% β-sheet and 9% ± 3.1% α-helix respectively) and 75% ± 3.1% of disordered conformations. These data are in agreement with the previous work by Song and co-workers^24^ and also with the estimated content of secondary structural elements based on the disorder predictor GlobPlot2.3^25^ and the available structural models (see Ref.^26^ and PDB 2LA6), which account for 11% β-sheet and 7% α-helix and 72% disordered polypeptide.

The full-length FUS protein has never been characterized in detail by NMR spectroscopy in solution since it is prone to aggregation, but the structure in solution of the RRM domain (residues 278–385, PDB 2LCW, and residues 282–370, PDB 2LA6) was determined^26^. We thus took advantage of the available sequence specific assignment (BMRB ID 17508) to assign the RRM signals in our experimental conditions by using a sub-set of NMR experiments (see “Methods”). The ^1^H-^15^N correlation map (Fig. 2B) is consistent with the spectra available in the literature for the well-dispersed signals of the RRM domain^24,26,27^. The small differences in chemical shift of the signals of our construct with respect to those of the isolated domain are due to differences in buffer composition, as the deposited chemical shift values can be matched by changing the sample conditions. Thus, interactions of the RRM domain with its flanking regions, if present, should be transient and not affecting the NMR signals. The signals of the disordered regions are clustered at the centre of the ^1^H-chemical shift region, with particularly high overlap in the so-called glycine region, as expected.

The dynamics of the isolated RRM domain has been already investigated by NMR spectroscopy^24^. We acquired and analyzed ^15^N relaxation properties of the RRM domain to investigate the conformational dynamics of the folded domain in our longer construct. The ^15^N longitudinal relaxation rate (^15^N R_1_) and the ^15^N transverse relaxation rate (^15^N R_2_) confirm the general features of the domain, without major variation of the ns-ps dynamics of the folded domain and with little changes consistent with an overall increase in correlation time induced by the presence of the additional residues of the two dynamical flanking regions (see Supplementary Fig. S1 online). The ^1^H-^15^N NOE experiments confirm the structured nature of the folded RRM domain, which maintains its overall compactness with a slight increase in dynamics in the region 305–320, in the β-hairpin region between α1 and β2, and in the region 325–335, which comprises the terminal part of β2 and extends to the β3 strand.

The two RGG boxes are completely disordered; EPR spectroscopy is therefore a suitable complement to NMR spectroscopy for obtaining information on their dynamics.

### Insights on FUS^165–422^ dynamics features through paramagnetic probes

EPR spectroscopy combined with site-directed spin labeling (SDSL) is a powerful method to investigate protein dynamics. From the analysis of the lineshape of the EPR spectrum of a spin label (i.e. nitroxide radicals) selectively grafted on an appropriate residue (i.e. cysteine, tyrosine, etc.) and the determination of its mobility, protein conformation can be characterized^28^. Sharp EPR signals are due to a fast tumbling rate of the spin label indicating a high degree of protein flexibility (small τ_C_ value), while a decreased mobility determines broader lines and higher τ_C_ values. This methodology has been extensively applied to characterize intrinsically disordered proteins and to follow possible disorder-to-order transitions^29,30^.

**Figure 3:**
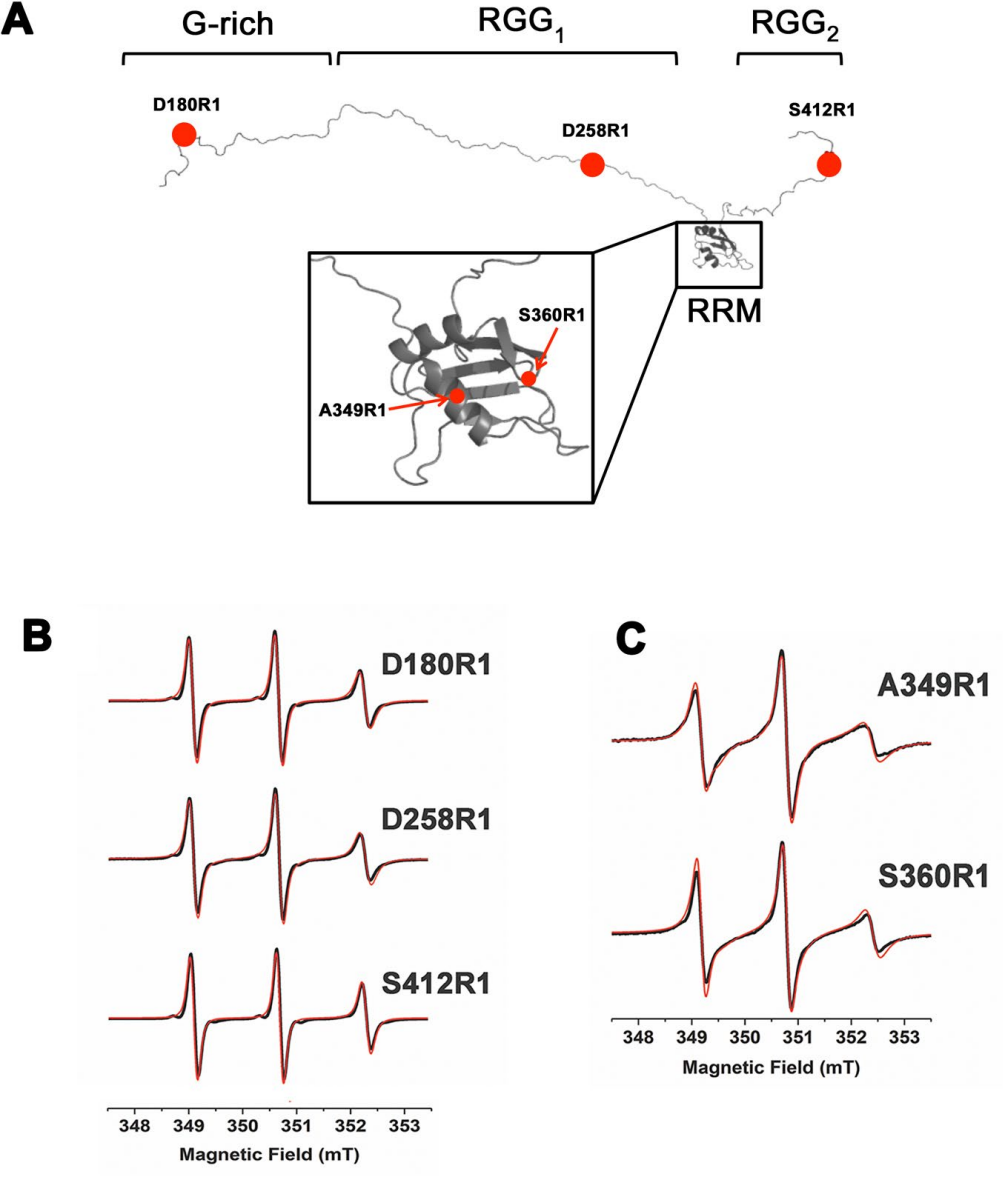
(**A**) Labelling positions selected for G-rich, RGG_1-2_ and RRM domains of FUS^165–422^. (**B**,**C**) Experimental (black line) and simulated (red line) X-band (9.8 GHz) CW-EPR spectra of D180R1, D258R1, S412R1, A349R1 and S360R1 spin labelled mutants of FUS^165–422^.

To monitor the structural behaviour of FUS, including the G-rich, the RGG_1_ and the RGG_2_ regions, as well as the RNA-binding site, five different mutants of FUS^165–422^ to insert a cysteine residue in the protein sequence were designed (see “Methods”). The choice of the positions was driven by the occurrence in the primary sequence of many glycine residues as well as by the presence of amino acids repeats (GRS) which limited the accessible mutation positions. In the end, we were able to mutate to cysteine the amino acids D180, D258, and S412. The two positions in the RRM domain, A349 and S360, were selected in a region opposite to the site for nucleotides interaction to avoid interference with the RNA-binding activity of FUS^165–422^ (Figs. 1B and 3A). Each of these mutants was labelled with MTSL (see “Methods”, the resulting mutants are marked R1) and X-band (9.8 GHz) CW-EPR spectra were recorded at room temperature (Fig. 3B,C).

The D180R1 and S412R1 variants, bearing the spin label in the G-rich and in the RGG_2_ region respectively, show a narrow EPR signal typical of a mobile stretch (Fig. 3B). The high mobility of these regions is confirmed by simulations of the spectra, which reveal the presence of a single spectral component with short τ_C_ value (0.22 ± 0.08 ns and 0.16 ± 0.08 ns respectively, Table 1). These results indicate that these two regions of FUS (G-rich and RGG_2_) have a large degree of fluctuations and lack a defined conformation. The CW-EPR spectrum of D258R1 (Fig. 3B) is instead composed by two different components: a sharp signal associated to a fast tumbling rate of the grafted spin probe (component 1 = 74 ± 3%, τ_C_ = 0.22 ± 0.08 ns) and a minor component with a higher τ_C_ value (component 2 = 26 ± 3%, τ_C_ = 1.60 ± 0.08 ns) (Table 1), revealing the occurrence of different dynamic states in solution. The broad component can be ascribed to restrictions induced by the proximity of the RRM domain to the tag position and to possible inter-domains interactions between these regions, considering the absence of significant signals’ linewidth broadening in the NMR spectrum of the mutant that can be associated to a conformation change due to the Asp to Cys substitution or to the presence of the MTSL tag (see PRE experiments below).

**Table 1:** Component percentages (%) and correlational times (τ_C_) calculated from simulations (SimLabel software, ref.^44^) for each spin labeled FUS^165–422^ mutants in the disordered domains, alone and in presence of hnRNPA2/B1 stem-loop RNA.

MTSL-lablled mutants	Component 1		Component 2	
	%	τ_C_ (ns)	%	τ_C_ (ns)
D180R1	100	0.22		
D180R1 + RNA	100	0.16		
D258R1	74	0.22	26	1.60
D258R1 + RNA	58	0.27	42	1.92
S412R1	100	0.16		
S412R1 + RNA	100	0.16		

Standard deviations on components percentages and τ_C_ were estimated equal to ± 3% and ± 0.08 ns respectively.

CW-EPR spectra were also recorded for the A349R1 and S360R1 mutants bearing the spin label on the α2 helix and on the flexible loop connecting α2 and β4 of the RRM domain, respectively (Fig. 3A). The EPR spectrum of A349R1 (Fig. 3C) is composed by three different components, indicating the coexistence of various dynamic states for the system (Table 2). In particular, two components indicate the presence of conformations with a high degree of flexibility (component 1 = 15% ± 4%, with τ_C_ = 0.40 ± 0.09 ns and component 2 = 50 ± 4%, with τ_C_ = 0.72 ± 0.09 ns) together with a conformation with reduced dynamics (component 3 = 35 ± 4% with τ_C_ = 1.40 ± 0.09 ns). Similarly, the EPR analysis of the S360R1 mutant (Fig. 3C) indicate that also this part of the RRM motif experiences multi-dynamic states. Interestingly, the broad EPR signal component (component 3 of S360R1) shows a τ_C_ value (2.17 ± 0.09 ns) higher than that determined for the same component in A349R1 (τ_C_ = 1.40 ± 0.09 ns). These differences in correlational time values between the two labelling positions could be caused by the spin label position. In fact, the less restricted environment for the label grafted on the α2 helix should confer a larger tumbling rate (smaller τ_C_) to MTSL compared to the S360R1 mutant.

**Table 2:** Component percentages (%) and correlational times (τ_C_) calculated from simulations (SimLabel software, ref.^44^) for each spin labeled FUS^165–422^ mutants in the RRM domain, alone and in presence of hnRNPA2/B1 stem-loop RNA.

MTSL-lablled mutants	Component 1		Component 2		Component 3	
	%	τ_C_ (ns)	%	τ_C_ (ns)	%	τ_C_ (ns)
A349R1	15	0.40	50	0.72	35	1.40
A349R1 + RNA	65	0.30			35	1.40
S360R1	72	0.48			28	2.17
S360R1 + RNA	27	0.57	35	0.78	38	2.13

Standard deviations on components percentages and τ_C_ were estimated equal to ± 4% and ± 0.09 ns respectively.

**Figure 4:**
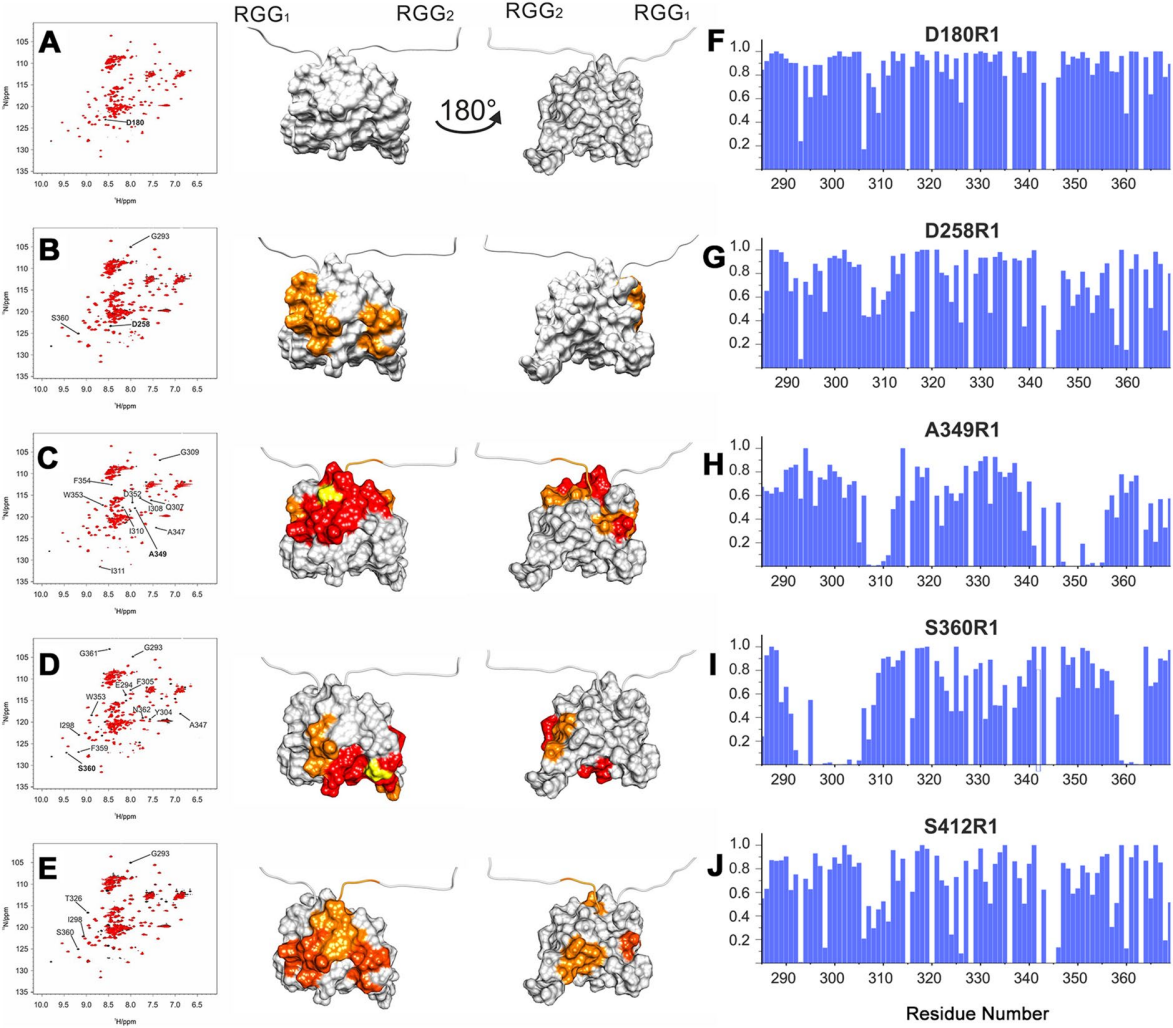
Left panels—^1^H-^15^ N HSQC NMR spectra of MTSL-labelled (red) and unlabelled (black) FUS^165–422^ mutants (**A**) D180R1, (**B**) D258R1, (**C**) A349R1, (**D**) S360R1 and (**E**) S412R1 with the assignment of the relevant cross peaks; Central panels—mapping on the protein surface of the RRM domain (PDB ID 2LA6) of the relevant changes in signal intensity. Residues are coloured from red to orange according to PRE magnitude (**F**–**J**); unaffected residues are coloured in grey. The labelled position in the RRM is coloured in yellow. Right panels: Paramagnetic relaxation enhancement values reported as normalized intensity ratio (I/I°) for FUS^165–422^ mutants (**F**) D180R1, (**G**) D258R1, (**H**) A349R1, (**I**) S360R1 and (**J**) S412R1.

In order to obtain further information on the influence of the RGG boxes on the RRM domain, we exploited Paramagnetic Resonance Enhancement (PRE) NMR experiments to determine possible long-range contacts between different protein regions^31,32^. Using the same five labelled mutants of FUS^165–422^ (D180R1, D258R1, A349R1, S360R1 and S412R1, Fig. 3A) previously characterized by EPR, we acquired pairs of ^1^H-^15^N-HSQC spectra of the MTSL-labeled and of the unlabeled mutants monitoring the effect of the unpaired electron of the spin label on signals broadening (Fig. 4).

PREs data obtained on D180R1 (Fig. 4A,F) confirm that this region of the G-rich domain is not in direct contact with RRM. The analysis of the PRE observed on the D258R1 (Fig. 4B,G) mutant shows some minor effects on the signals of residues in the 306–311, 342–346 and 357–360 regions, suggesting the occurrence of some transient interactions between RGG_1_ and the RRM domain. On the RGG_2_ side, the paramagnetic effects produced by the label in position S412R1 (Fig. 4E,J) are comparable to the ones of D258R1 mutant and affect several positions on the protein surface confirming the high flexibility of this harm.

The same PRE NMR experiments were acquired for the A349R1 and S360R1 mutants. The presence of a spin label on the α_2_ helix bearing A349R1 cause severe broadening of the signals of a stretch encompassing residues 308–312 (Fig. 4C,H), which is a loop juxtaposed to the α_2_ helix, and a slight effect on some signals of residues located in the strand immediately preceding the helix and towards the end of the RRM domain. The effect of the spin label on S360R1 (Fig. 4D,I) results in the selective broadening of the signals of two regions: the region around the paramagnetic tag (residues 357–364) and a region inside the RRM domain encompassing residues 291–308. The PRE pattern reflects the spatial proximity of the label to the hairpin between β_1_ and α_2_ and the helix itself; the extension of this segment underlines the high dynamics of the region.

### Interaction of FUS^165–422^ with stem-loop RNA

In order to test the capability of FUS^165–422^ to interact with RNA, we performed experiments in the presence of hnRNPA2/B1 stem-loop. Since the RRM of FUS binds a NYNY single strand (N = C or U, Y = A, C, G and U)^27^, we performed all the spectroscopic measurements with a RNA sequence containing these specific nucleotide quartets. Previous studies measured the affinity of the full length FUS and various protein segments for different nucleotide sequences, revealing that the RGGs motifs strongly influence the capability of the protein to bind RNAs^18^.

**Figure 5:**
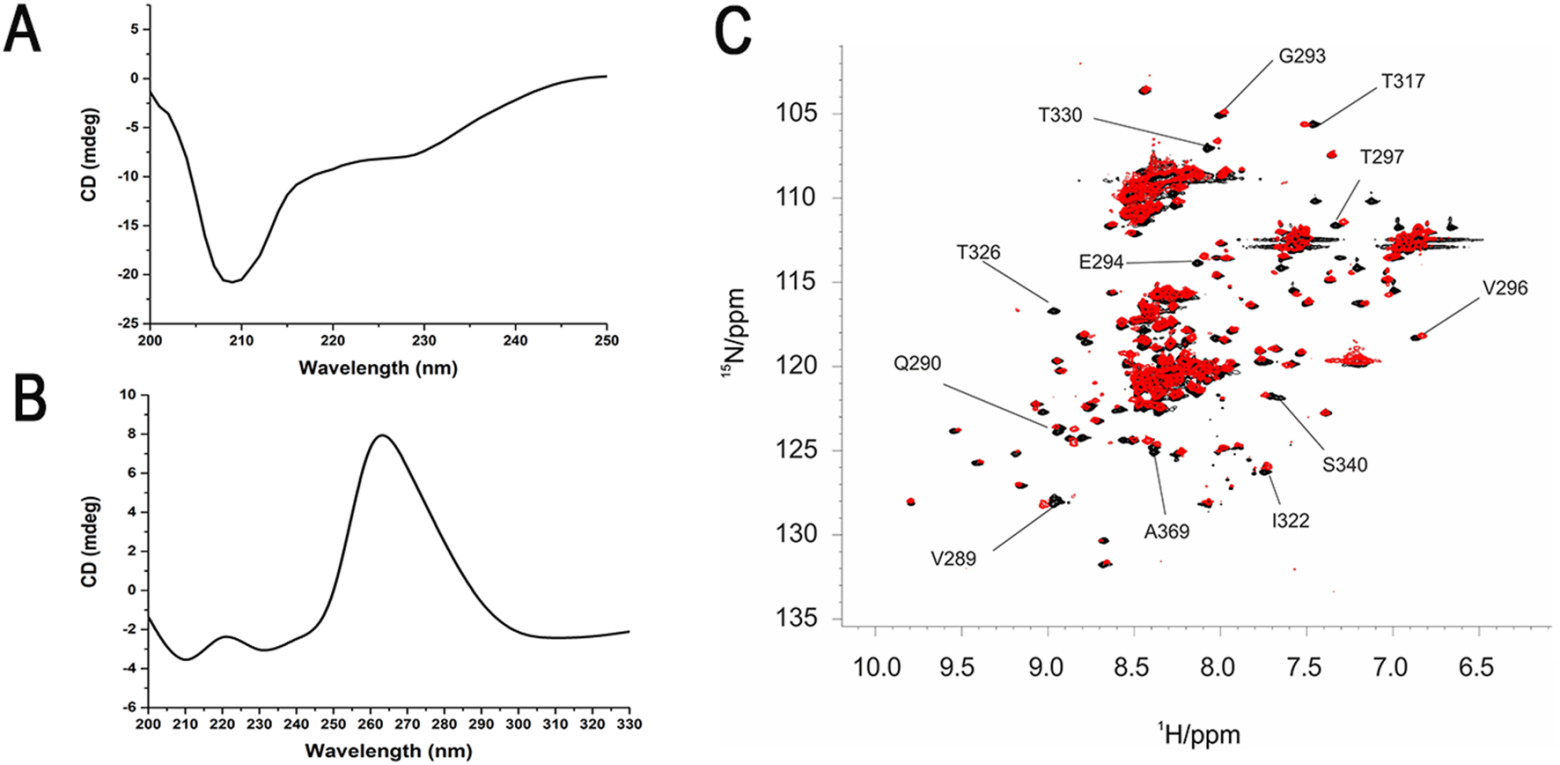
(**A**) Far-UV CD spectra (200–250 nm) of FUS^165–422^ in the presence of hnRNPA2/B1 stem-loop RNA; (**B**) Far-UV CD spectra (200–250 nm) of hnRNPA2/B1 stem-loop RNA; (**C**) 2D ^1^H-^15^N HSQC spectrum of FUS^165–422^ in the presence of hnRNPA2/B1 stem-loop RNA (red) superimposed with the spectrum of the free protein in solution (black) with the assignment of selected cross peaks.

Upon addition of hnRNPA2/B1 stem-loop RNA to FUS^165–422^, at 1:1 molar ratio, no major changes in the structure of the protein occur. The far-UV CD spectrum showed a minor shift of the maximum absorbance band (from 207 to 210 nm) and a weak variation in the spectral lineshape (Fig. 5A), suggesting the absence of a major structural reorganization upon RNA-binding. The far-UV CD spectrum of stem-loop RNA alone is shown in Fig. 5B.

Consistently with the CD spectrum, ^1^H-^15^N correlation NMR spectrum does not show major variations due to conformational changes occurring upon RNA interaction (Fig. 5C). The comparison with the free-form spectrum shows several chemical shift changes due to RNA binding for signals of residues in the RRM domain. The largest changes occur in the regions around residues 286, 326 and 369, confirming the location of the binding site in the proximity of these amino acids^27^. The spectral features of the disordered regions of the two RGG boxes were instead unchanged. We cannot rule out a fuzzy interaction that cannot be detected by NMR experiments, but RNA binding does not produce any stable conformational change in the disordered parts of FUS^165–422^.

The CW-EPR spectra of D180R1 and S412R1 do not show any relevant variations in the spectral lineshape (see Supplementary Fig. S2 online) and the τ_C_ value (Table 1) in the presence of RNA, indicating the absence of a direct interaction of the G-rich and the RGG_2_ regions with nucleotides.

**Figure 6:**
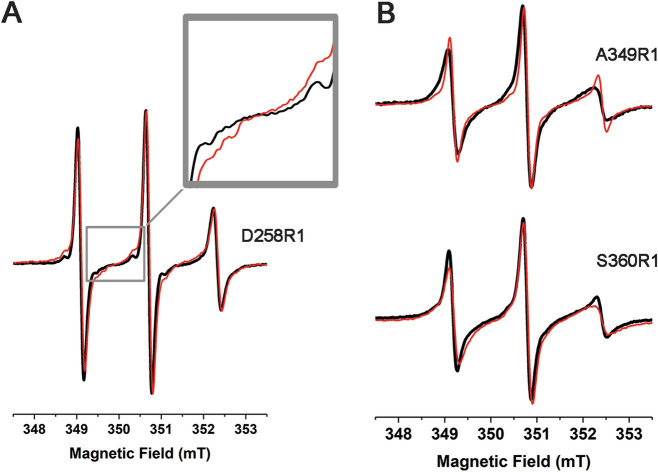
X-band (9.8 GHz) CW-EPR spectra without (black line) and in the presence of 1:1 hnRNPA2/B1 stem-loop RNA (red line) recorded spin labelled FUS^165–422^ mutants (**A**) D258R1 and (**B**) A349R1 and S360R1.

On the other hand, the broadening of the EPR spectrum of the D258R1 labelled mutant (Fig. 6A) and the variation in the ratio of the components constituting the signal suggest an interaction of the RGG_1_ region with the nucleotides. However, the increase in τ_C_ for the broader component is small (Δτ_C_ = 0.32 ns Table 1) suggesting that the variation in RGG_1_ dynamics is also small and does not induce a relevant structural effect.

More dramatic effects occurred in the RRM region. For A349R1, the narrowing of the corresponding EPR lines (Fig. 6B) indicates fast motion of the α_2_ helix, which surprisingly increases in the presence of stem-loop RNA. The effect on the dynamic of this FUS region induced by nucleotides interaction appears also observing the increase of the sharpest component percentage (from 15 ± 4% in solution to 65 ± 4%, with stem-loop RNA, Table 2). Since the tumbling of the RRM domain is influenced by its transient interactions with the RGG boxes, as revealed by CW-EPR and NMR experiments, the enhancement in dynamics suggests a reduction in these interactions upon RNA binding.

In the presence of RNA the EPR spectrum of S360R1 (Fig. 6B) results broader than the one recorded in its absence. The simulation suggests significant differences in the spectral parameters mainly for component 1, but also the appearance of a new sharp component (component 2, see Table 2).

To get insights on the structural variations of FUS^165–422^ upon RNA interaction, Q-band DEER experiments were performed. This pulse-EPR technique permits to measure the distance between multiple spin probes attached to a biomolecule in various experimental conditions^28,30,33^. Two different FUS^165–422^ mutants bearing two cysteine residues were produced and labeled with MTSL (D258R1/A349R1 and A349R1/S412R1). On each of these mutants the 4-pulse Q-band DEER traces were recorded in the absence and in the presence of stem-loop RNA to understand the spatial arrangement of the RGG boxes compared to the RRM region and to identify their role in the RNA interaction process.

**Figure 7:**
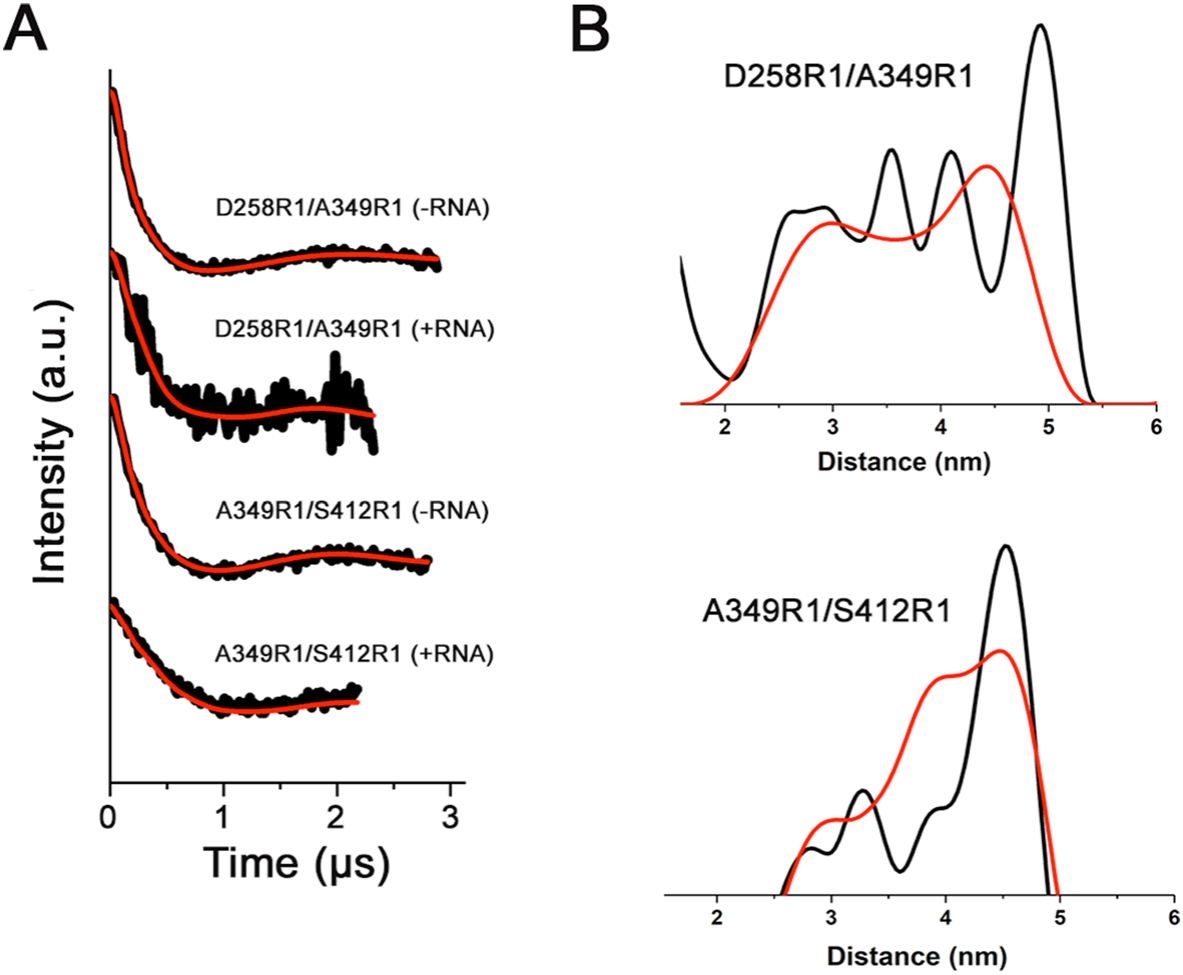
(**A**) Background corrected Q-band (9.8 GHz) DEER traces (black line) and corresponding fitted curve (red line) for D258R1/A349R1 and A349R1/S412R1 mutants of FUS^165–422^ in without and in the presence of hnRNPA2/B1 stem-loop RNA; (**B**) corresponding distance distribution without (black line) and in the presence of stem-loop RNA (red line) recorded for D258R1/A349R1 (top panel) and A349R1/S412R1 (bottom panel) mutants of FUS^165–422^.

Background corrected DEER time traces, recorded for both doubly labelled mutants with and without RNA, are reported in Fig. 7A. The inter-spin distances for D258R1/A349R1 and A349R1/S412R1 mutants in the absence of RNA (Fig. 7B black lines, top and bottom panel respectively) showed a similar profile for both samples with peak maxima at 5.0 nm and 4.7 nm Å (± 0.4 nm), respectively. The broad profiles of distributions is related to the large mobility of the RGG regions in solution due to their intrinsically disordered behaviour.

Upon RNA interaction, changes on spin–spin distances distributions are mainly observed for the D258R1/A349R1 sample (Fig. 7B red line, top panel), even if the DEER traces present a lower signal-to-noise ratio than that recorded in absence of nucleotides. In this case, a shift to lower distances (from 5.0 to 4.3 nm) of the maximum peak occurred, highlighting a weak rearrangement of RGG_1_ and RRM induced by RNA binding. Analogues Q-band DEER experiments performed on the A349R1/S412R1 system with stem-loop RNA (Fig. 7B red line, bottom panel) reveal a very broad inter-spin distance distribution. A less evident main peak compared to that obtained in the absence of nucleotides is present in the range ~ 4–5 nm. These data suggest that both RGG regions do not undergo significant conformational changes in the presence of stem-loop RNA, keeping their native disordered behaviour in the presence of nucleobases.

## Discussion

RGG motifs are continuous Arg-Gly-Gly repeats in the primary structure of different types of RBPs and this unique amino acid composition seems to be a key factor in the mechanisms of protein-RNA and/or protein–protein interaction.

The FUS protein possesses three different RGG domains; the C-terminal RGG_3_ domain has been well characterized and demonstrated to be important for the interaction with Transportin, in phase-separation and stress granule formation of FUS^19–21^. RGG_1_ and RRG_2_ regions were independently characterized in constructs including the RRM but they were never characterized together with the intervening RRM domain. We thus decided to focus on the FUS^165–422^ construct comprising both the RGG_1_ and RGG_2_ regions, and on their interaction with RNA.

The RGG regions have been predicted to be completely disordered thus with a high degree of flexibility^15,17,18,27^. Far-UV CD and NMR experiments performed on FUS^165–422^ confirmed that only part of the polypeptide is structured. In addition, X-band CW-EPR, DEER and NMR experiments on different spin labeled FUS variants revealed the great mobility of the G-rich, RGG_1_, and RGG_2_ regions typical of IDPs, as previously suggested^26,34^. IDPs can be characterized by ensembles of rapid interconverting extended and compact conformations of protein stretches^35^. This definition perfectly fits with the properties of the RGG_1_ and the RGG_2_ regions. The DEER inter-spin distances obtained for doubly MTSL-labelled FUS^165–422^ mutants have a broad distribution, suggesting that both motifs simultaneously possess both elongated and compact forms in solution. These concomitant “open” and “close” states have been also found in TDP-43, which is a protein related to FUS. NMR spectroscopy showed that TDP-43 has dynamic inter-domains interactions mediated by IDRs that could control the functions and the proteinopathy of the protein^36^.

We can speculate that RGG_1_ and RGG_2_ interact with RRM, due to the multimodal and broad shape of the DEER inter-spin distance profile. However, these contacts between domains should be transient interactions in the ps-ns range since NMR experiments recorded for FUS^165–422^ mutants bearing MTSL in RGG boxes did not reveal strong PRE effects on the RRM domain. This is also in agreement with CW-EPR data reflecting that only a small fraction of the RGG_1_ conformation ensembles possessed partial restrict motions due to steric hindrance exerted by the globular domain.

The RGG regions result particularly important for the physiological RNA-binding mechanism of FUS. Even if the main sites for RNA interaction of FUS are the RRM and the ZnF domains, the RGG repeating motifs coupled to adjacent folded domains showed affinities approaching those of full-length FUS, suggesting that the RGG boxes might act as primary mediators in the nucleotide binding process^18^. In fact, Allain and co-workers recently assessed that the RGG_2_ domain of FUS is implicated in stem-loop RNA structural destabilization favoring the interaction of the RRM. In terms of dynamics, no evident change upon RNA interaction was detected by CW-EPR experiments for the RGG_2_ domain suggesting that this region of FUS does not undergo any conformational variation in the presence of RNA. This behaviour was also supported by Q-band DEER measurements since a broad shape of inter-spin distance distributions was observed for the A349R1/S412R1 system with and without RNA, evidencing that the RGG_2_ motif could retain its flexibility with the hnRNPA2/B1 stem-loop in solution. Considering that various studies observed a synergistic RNA-binding mechanism of protein domains, this discrepancy of results could be related to the absence of the ZnF domain in the FUS^165–422^ construct used here^17,27^.

Although the RGG_1_ motif resulted to be very flexible, it displays some small variations upon RNA-binding compared to the other disordered regions of FUS^165–422^. In particular, partial decrease in mobility was detected for stem-loop RNA binding by CW-EPR while DEER experiments indicated a minimal reduction in distances between RGG_1_ and the RRM domain. These findings could be related to the formation of a less-extended conformation (i.e. β-turns) during RNA-coordination, as previously determined for others RRG motifs of FUS and similar proteins^34,37,38^. However, no drastic variations have been observed for RGG_1_ in complex with RNA in this study thus we can infer that also this IDR of FUS does not undergo a defined disorder-to-order transition in presence of nucleotides. Summarizing, the experiments performed in this work were not able to detect any direct interaction of the RGG regions with stem-loop RNA.

Recently, several studies evidenced that a large class of proteins that contain IDR connecting globular domains form fuzzy complexes with partners (i.e. protein or nucleic acids) with different topologies^39^. Flanking models are a category of complexes that lack a defined conformation where IDRs increase the propensity of the folded regions toward binding of competent form and regulate the entropy of the process^40^. We speculate that this type of conformational model could be adopted by RGG_1_ upon coordination of stem-loop RNA, as also previously demonstrated for another splicing factor complex (U2AF^65^/SF1) sharing various similarities with the FUS protein^41^.

Various RBPs have evolved a great variety of pathways to remodel RNA. These proteins, which generally contains a large number of cationic residues, transiently interact with nucleotides enabling the RNA backbone to assume wide conformations^42^. Moreover, Allain and co-workers speculated about the role exerted by the RGG regions of FUS in RNA destabilization and remodelling, without assuming a folded conformation^27^. Thus, the possibility that RGG_1-2_ of FUS protein could bind transiently the hnRNPA2/B1 stem-loop RNA cannot be ruled out.

The presence of the adjacent RGG motifs significantly increases the affinity of the RRM domain for different nucleotide sequences, and structured RNA in particular^18,27^. We thus investigated the structural effects of stem-loop RNA coordination on the RRM region of FUS. In solution, this domain has a more compact conformation compared to RGG_1_ and RGG_2_ but it experiences a more pronounced mobility than generally observed for a normal globular protein^43^. Based on CW-EPR spectra recorded in the presence of RNA, the RRM domain revealed concomitant increase and a decrease of local flexibility, underlining that remodelling of the conformation takes place upon nucleotide binding. This capability to modulate its structure, in combination with the RNA destabilization exerted by RGG motifs, supports the recent speculation that RRM nucleotide recognition is more based on RNA shape than on nucleobase sequence. However, further studies are needed to confirm this finding.

Summarizing, our results provide additional structural information on the RGG domains of FUS and their implication in RNA binding activity. Through an integrated approach of EPR and NMR spectroscopies, the degree of disordered of the RGG boxes of FUS have been characterized even if future experiments aimed to individuate their role in RNA-binding are necessary. The experimental data revealed also a dynamic effect of these flanking domains exerted on the folded region and a variation in terms of terms of tumbling rates for the α_2_ helix and the flexible loop connecting α_2_ and β_4_ of the RRM domain during stem-loop RNA coordination. RGG motif should represent a key-factor for determining the physiological and also pathological pathways of different human proteins related to fatal neurodegenerative diseases, such as ALS and FTLD. Further biochemical and biophysical efforts on the characterization of the role exerted by these domains in protein activities should be performed in order to contribute to the development of possible therapies for the treatment of the related pathologies.

## Methods

### Protein expression and purification

The gene encoding for FUS^165–422^ fragment (GENEWIZ) was cloned in the pENTR vector for the Gateway cloning technology and subsequently in the pDEST-HisMBP vector (which adds a His tag fused with the Maltose Binding Protein at the N-terminus of the protein), utilizing the pENTR/TEV/D-TOPO cloning kit (Invitrogen). *E.coli* BL21 (DE3) cells were transformed with the plasmid pDEST-HisMBP-FUS^165–422^, grown in LB medium (or in ^15^N M9 medium for NMR experiments) to mid-log phase at 37 °C, induced with 0.50 mM IPTG, and then they were kept at 20 °C for at least 16 h. For protein purification we adapted the protocol established by Ozdilek et al.^18^. In brief, the cells were harvested and re-suspended in 50 mL of binding buffer (20 mM sodium phosphate buffer at pH 6.5, 5 mM of DTT, 5 mM of Imidazole, 10% v/v of glycerol and 1.5 M of NaCl) supplemented with protease inhibitor tablets (Bayer) and successively lysed by sonication. The lysate was passed through a 5 mL HisTrap FF affinity column (GE Healthcare Life Sciences) and washed with the elution buffer (20 mM sodium phosphate buffer at pH 6.5, 5 mM DTT, 1.5 M of NaCl, 500 mM imidazole and 10% v/v of glycerol). The His-MBP tag was cleaved incubating the protein solution overnight with TEV protease and successively separate from the fusion protein with a second step of purification with 5 mL HisTrap FF affinity column using the same elution buffer reported above.

As final purification step, a gel filtration using a HiLoad 16/600 Superdex 200 column (GE Healthcare Life Sciences) was performed to separate FUS^165–422^ from possible contaminants and to transfer the protein in the final buffer (20 mM MES at pH 6.5 with 150 mM NaCl). The corresponding MALDI-TOF spectrum (see Supplementary Fig. S3A online) was acquired as control, reporting the peak at about 25,000 m/z, corresponding to the correct molecular weight of FUS^165–422^. In addition a SDS-page gel was performed to check the purity of the protein at the end of the purification protocol (see Supplementary Fig. S3B online).

### Site directed mutagenesis for cysteine mutants

The five single cysteine mutations (D180C, D258C, A349C, S360C and S412C) were inserted into FUS^165–422^ sequence using the kit Quick Change II Site-Directed Mutagenesis Kit (Agilent). The primers of each variant (see Supplementary Table S4 online) were placed in solution with pENTR-FUS^165–422^ vector and successively subjected to 18 steps of PCR reaction. The final products, after DNA sequencing controls, were cloned into pDEST-HisMBP and the relative protein variants were produced and purified as described previously.

The plasmids encoding the protein bearing the double cysteine variations (D258C/A3494C and A349C/S412C) were obtained as described previously using the pENTR-FUS^165–422^ vector carrying already the A349C mutations and then introducing D258C and S412C mutations respectively.

### Site-directed spin labeling reaction

Each protein solution, containing ~ 100 nmol of FUS^165–422^ single and double cysteine variants, was incubated with a tenfold molar excess of DTT at room temperature in order to reduce the cysteine thiol group. After 30 min, the reaction mixture was eluted into a PD-10 desalting column to remove the reducing agent using MES buffer (20 mM MES at pH 6.5 with 150 mM NaCl) as mobile phase. Successively S-(1-oxyl-2,2,5,5,-tetramethyl-2,5,-dihydro-1H-pyrrol-3-yl) methylmethane-sulfonothiolate (MTSL) was added to the solution with 1:10 protein:label molar ratio; the reaction was kept at room temperature under gentle stirring and in absence of light for at least 10 h. The unreacted MTSL was removed using a PD-10 desalting column using MES buffer. The fractions containing single (D180R1, D258R1, A349R1, S360R1 and S412R1) and double (D248R1/A349R1 and A349R1/S412R1) MTSL-labelled positions of FUS^165–422^ were individually checked recording the relative X-band CW-EPR spectrum, to assure the complete removal of the free tag. Successively all the fractions containing labelled samples were pooled together and the solution was concentrated using AMICON ultra centrifugal filters (MWCO = 10 kDa). The final protein concentration was calculated observing the absorbance at 280 nm (ε = 17,420 M^-1^ cm^-1^) with a UV/Vis Varian Cary 50 spectrophotometer. The labelling yield of each sample (see Supplementary Table S5 online) was calculated from the double integral of CW-EPR spectrum and it was compared with a calibration curve obtained for free MTSL in water solution at different molar concentrations.

For PRE experiments, the FUS^165–422^ mutants bearing cysteine substitution without MTSL grafted on the thiol group were used as diamagnetic samples.

### hnRNPA2/B1 stem-loop RNA preparation

hnRNPA2/B1 stem-loop RNA (GGCAGAUUACAAUUCUAUUUGCC) was synthetized by Eurofins Genomics. The lyophilized product has been dissolved in RNAase free buffer (20 mM MES at pH 6.5 and 150 mM NaCl) and the concentration of stock solution was checked measuring the absorbance at 260 nm with UV/vis spectroscopy. For CD, EPR and NMR analysis of FUS^165–422^ in presence of hnRNPA2/B1 stem-loop RNA, the buffer solution containing the protein has been prepared in RNAase free solution in order to avoid possible RNA degradation.

### Circular Dichroism spectroscopy

Far-UV Circular Dichroism (CD) experiments were performed at room temperature with a Jasco CD-J-815 spectropolarimeter using a quartz cuvette with a path length of 1 mm length. CD spectrum of FUS^165–422^ in solution was recorded with a final protein concentration either of 15 μM or 50 μM in 20 mM of MES buffer (pH 6.5) and 150 mM of NaCl from 200 to 250 nm. At wavelength below 200 nm the absorbance due to the presence of NaCl, necessary for protein stability, saturate the photomultiplier and the data were discarded. Experiments in the presence of RNA were recorded placing in solution the protein with 1:1 FUS: hnRNPA2/B1 RNA molar ratio. Each spectrum was accumulated at least 10 times in order to improve signal-to-noise ratio. Secondary structure elements of the protein in different conditions were derived from CD spectra using Dichroweb analysis tool^23^. The prediction of the percentage of folded and disordered region based on the primary sequence of FUS^165–422^ were realized through the PDB structural model of the RRM (PDB ID 2LA6) and GlobPlot 2.3 program^25^.

### X-band CW-EPR spectroscopy

Each CW-EPR spectrum of MTSL-labeled FUS^165–422^ variants (D180R1, D258R1, A349R1, S360R1 and S412R1) in solution and with RNA was recorded with an ELEXYS E580 spectrometer equipped with a Super High Q resonator (SHQE) operating at X-band (9.8 GHz) at room temperature. Each CW-spectrum for spin labeled FUS^165–422^ variants were obtained with a final protein concentration of approximately 60 μM in 20 mM of MES buffer (pH 6.5) and 150 mM of NaCl. For the experiments in the presence of hnRNPA2/B1 RNA, a 1:1 protein:RNA ratio was used.

The following spectroscopic parameters were applied to record each spectrum: microwave power = 10 mW; magnetic field amplitude = 1G; field sweep = 150 G; receiver gain = 60 dB; modulation frequency = 100 kHz.

All EPR spectra were successively simulated with the program SimLabel (a GUI of Easyspin software)^44^ in order to obtain spectral component percentages and the corresponding correlational times (τ_C_). The error on the components’ percentages and the correlational times values for each spectrum were obtained performing three independent simulations of the same spectra. The correlation times and the component percentages reported in the text for each labelling position represent the average values arising from the spectral simulations.

### Q-band DEER spectroscopy

DEER experiments on FUS^165–422^ doubly spin labeled mutants (D258R1/A349R1 and A349R1/S412R1) in buffer solution (20 mM MES at pH 6.5 with 150 mM of NaCl) or in the presence of 1:1 protein:RNA were performed on Elexys E580 spectrometer coupled with the standard resonator EN5107D2 operating at Q-band (34 GHz) and a 3 W microwaves amplifier (see Supplementary Fig. S6 online). The spectrometer was equipped with an Oxford helium temperature regulation unit and the relative experiments were carried out at 50 K. Each sample was prepared with a final protein concentration of ~ 80–90 μM in the presence of glycerol (final concentration equal to 10% v/v).

DEER experiments were performed using the four-pulse dead-time free DEER sequence (π/2–π–π_PUMP_–π) setting π/2 = 20 ns and π = 40 ns . The inter-pulse t1 was set to 200 ns and the t2 was adjusted based on the phase memory constant time recorded for each labelled mutant. The pump ELDOR pulse (π_PUMP_ = 36 ns) was positioned at the center of the MTSL absorption spectrum and the frequency difference between the pump and the observer pulses was 65 MHz. The DEER traces were accumulated at least 14 h in order to minimize the signal to noise ratio.

DeerAnalysis 2019 software was used for the correction of background echo decay and consequently to obtain the inter-spin distances involving the Tikhonov regularization. The regularization factor (α) was selected into the corner, according to L-curve criteria.

### NMR spectroscopy

The 2D ^1^H-^15^N HSQC NMR spectra of a 100 μM ^15^N-FUS alone or in complex with hnRNPA2/B1 stem-loop RNA (1:1 FUS:RNA) were acquired at 298 K with a 22.3 T Bruker Avance III NMR spectrometer operating at 950.06 MHz for ^1^H and equipped with a cryogenically cooled triple resonance probehead (TCI).

Each 2D ^1^H-^15^N HSQC NMR spectrum of unlabelled and MTSL-labelled ^15^N-FUS^165–422^ variants (D180R1, D258R1, A349R1, S360R1 and S412R1, 200 μM) in solution were acquired at 298 K with a 22.3 T Bruker Avance III NMR spectrometer operating at 950.06 MHz for ^1^H and equipped with a cryogenically cooled triple resonance probehead (TCI).

All the heteronuclear relaxation experiments (R_1_, R_2_ and ^1^H-^15^N NOEs) were acquired using ^15^N labeled FUS at about 200 µM. The spectra were recorded at 298 K on a 16.4 T Bruker Avance NEO spectrometer operating at 700.06 MHz for ^1^H and equipped with a cryogenically cooled triple resonance probehead (TCI). The ^15^N R_1_ and R_2_ experiments were acquired with 8 scans per increment (2048 × 320 points). To determine the ^15^N R_1_ the following delays were used: 20 ms, 60 ms, 120 ms, 180 ms, 240 ms, 400 ms, 600 ms, 800 ms, 1 s, 1.2 s. To determine the ^15^N R_2_ the following delays were used: 32 ms, 64 ms, 128 ms, 160 ms, 190 ms, 220 ms, 260 ms, 320 ms, 360 ms, 440 ms, 500 ms. The relaxation delay for ^15^N R_1_ and R_2_ experiments was 3.0 s. The ^1^H-^15^N NOEs experiments were acquired with 64 scans (2048 × 320 points) and a relaxation delay of 6.0 s, used for ^1^H saturation when necessary.

NMR data sets were processed using either Bruker TopSpin 3.5pl7 or TopSpin 4.0.6 software. CARA^45^ was used to analyze and annotate the spectra.

The ^15^N relaxation rates (R_1_ and R_2_) were determined by fitting the cross-peak intensity measured as a function of variable delay, to single-exponential decay using the Bruker Dynamics Center 2.4, available as stand-alone ancillary software of TopSpin by Bruker. The error bars are generated by the software using the peak intensities under the assumption that their uncertainties are unknown but equal for all Y values and the non-linear fit determines errors for the fitted parameters.^1^H-^15^N NOEs values were obtained as a ratio between peak intensity in spectra recorded with and without ^1^H saturation. The error can be estimated about 10% measuring the noise level of the spectrum without saturation.

The secondary structure propensity (SSP, see Supplementary Fig. S1 online) from heteronuclear chemical shifts was calculated by using the neighbour corrected structural propensity calculator (ncSPC) tool^46^ available online at http://nmr.chem.rug.nl and the BMRB 17508 data.

Chemical shift mapping was depicted on the RRM structure (PDB ID 2LA6) by using UCSF Chimera^47^, a molecular graphics program developed by the Resource for Biocomputing, Visualization, and Informatics at the University of California, San Francisco.

## Supplementary information

Supplementary Information
