# Supplementary material for: A combined NMR and EPR investigation on the effect of the disordered RGG regions in the structure and the activity of the RRM domain of FUS
Source: Sci Rep. 2020 Dec 1;10:20956. doi: 10.1038/s41598-020-77899-x (PMC7708983; doi:10.1038/s41598-020-77899-x)
Supplement: Supplementary file 1 — Supplementary Information [file 41598_2020_77899_MOESM1_ESM.pdf]

A combined NMR and EPR investigation on the effect of the disordered RGG regions in the structure and the activity of the RRM domain of FUS

A. Bonucci [a,b], M.G. Murrali [a], L. Banci [a,b], R. Pierattelli [a,b]

[a] CERM – Magnetic Resonance Center, University of Florence, Via Luigi Sacconi 6, 50019 Sesto Fiorentino, Italy

[b] Department of Chemistry, University of Florence, Via della Lastruccia 3, 50019 Sesto Fiorentino, Italy.

SUPPLEMENTARY MATERIAL

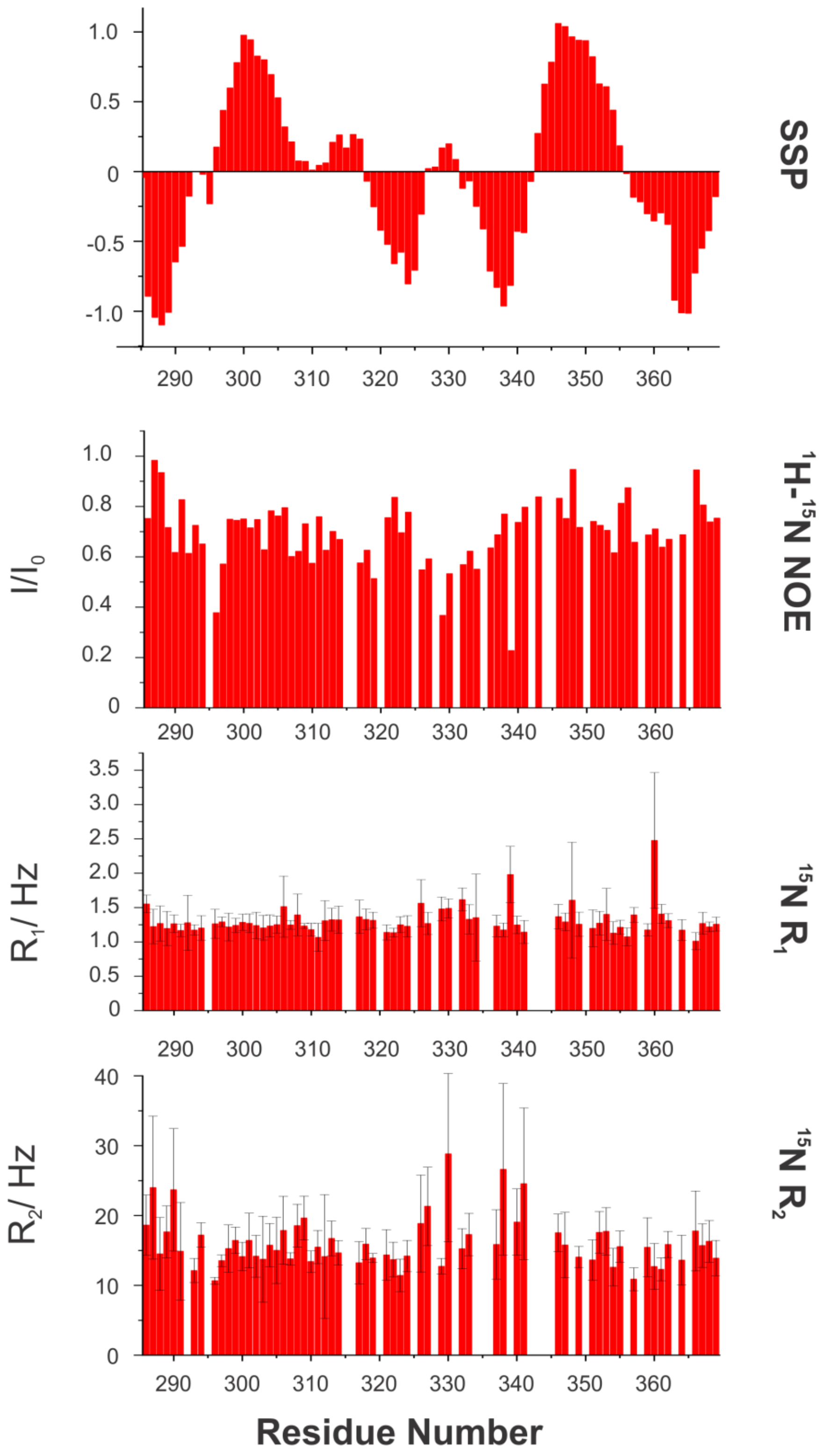

Supplementary Fig. S1: Heteronuclear relaxation data (^{1}H-^{15}N NOE, ^{15}N longitudinal relaxation rates R_1 and ^{15}N transverse relaxation rates R_2) for residues in the RRM region of FUS$^{165-422}$. In the upper panel the SSP plot derived from NMR chemical shifts is also reported to account for the presence of secondary structural elements.

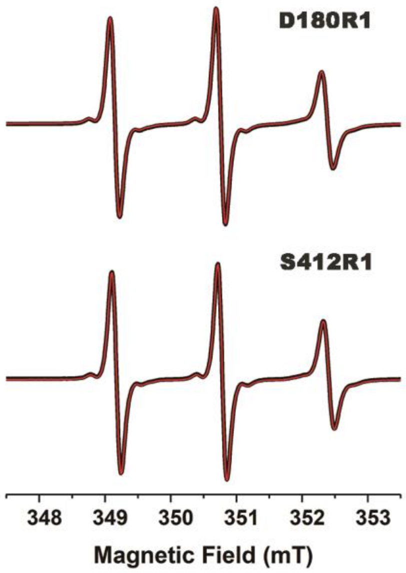

Supplementary Fig. S2: X-band CW-EPR spectra of D180R1 and S412R1 samples in the absence (black line) and in presence of stem-loop RNA (1:1 ratio)

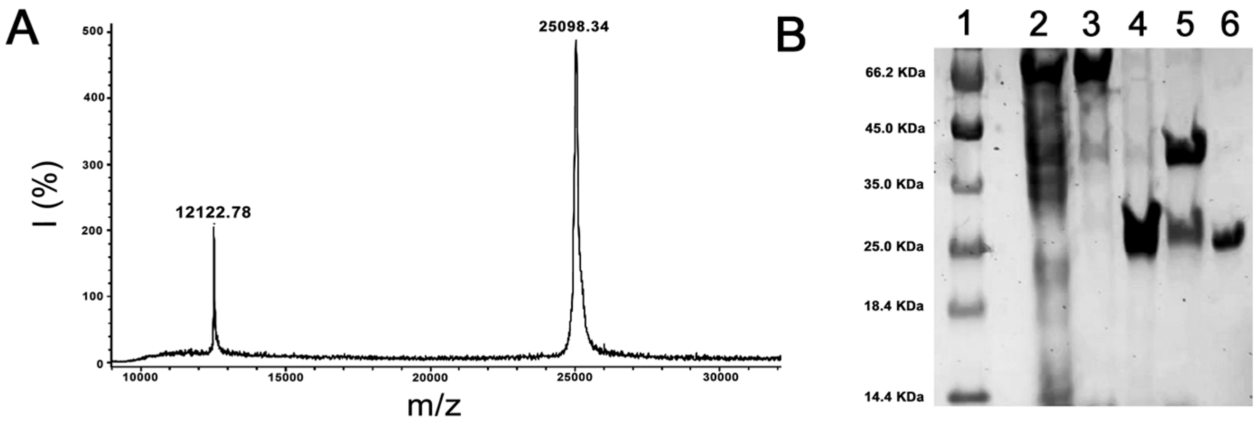

Supplementary Fig. S3: A) MALDI-ToF analysis for the determination of purity of RGG_1-RRM-RGG_2 fragment in 20 mM MES at pH 6.5 after purification. B) SDS-page performed for the purification of $FUS^{165-422}$: marker (1); cell extract containing His-MBP-$FUS^{165-422}$ (2); first step of His-MBP-$FUS^{165-422}$ purification using HisTrap FF affinity column (3); second step of $FUS^{165-422}$ purification using HisTrap FF affinity column after TEV proteolysis (4); control of TEV proteolysis of $FUS^{165-422}$ from His-MBP fusion protein (5); final purification step using a HiLoad 16/600 Superdex 200 column (6).

	**PRIMERS**
**D180C (forward)**	GCCGGTGGTAATTATGGCCAATGTCAGAGCAGCATGAGC
**D180C (reverse)**	GCTCATGCTGCTCTGACATTGGCCATAATTACCACCGCC
**D258C (forward)**	CGGTATGGGTGGAGCTGTCGTGGCGGCTTTAAC
**D258C (reverse)**	GTTAAAGCCGCCACGACAGCTGCCACCCATACCG
**A349C (forward)**	GATCCGCCGAGCCCAAATGCGCCATCGATTGGTTTG
**A349C(reverse)**	CAAACCAATCGATGGCGCATTTGGCGCTCGGCGGATC
**S360C (forward)**	GATCGGGTTGCCGCAGAACTCTTTGCCGT
**S360C (reverse)**	ACGGCAAAGAGTTCTGCGGCAACCCGATC
**S412 (forward)**	CGCCACCACCGCACGGAAAACCACC
**S412 (reverse)**	GGTTGGTTTTCCGTGCGGTGGTGGCG

Supplementary Tab. S4: Primer sequence used in site-directed mutagenesis reaction in order to obtaine $FUS^{165-422}$ single or double cysteine variant for cw-EPR and DEER experiments

**MTSL-labelled mutants**	**Labelling yield (%)**
D180R1	70
D258R1	72
A349R1	69
S360R1	60
S412R1	81
D258R1/A349R1	43
A349R1/S412R1	41

Supplementary Tab. S5: MTSL labelling yield calculated from X-band EPR spectrum of $FUS^{165-422}$ mutants

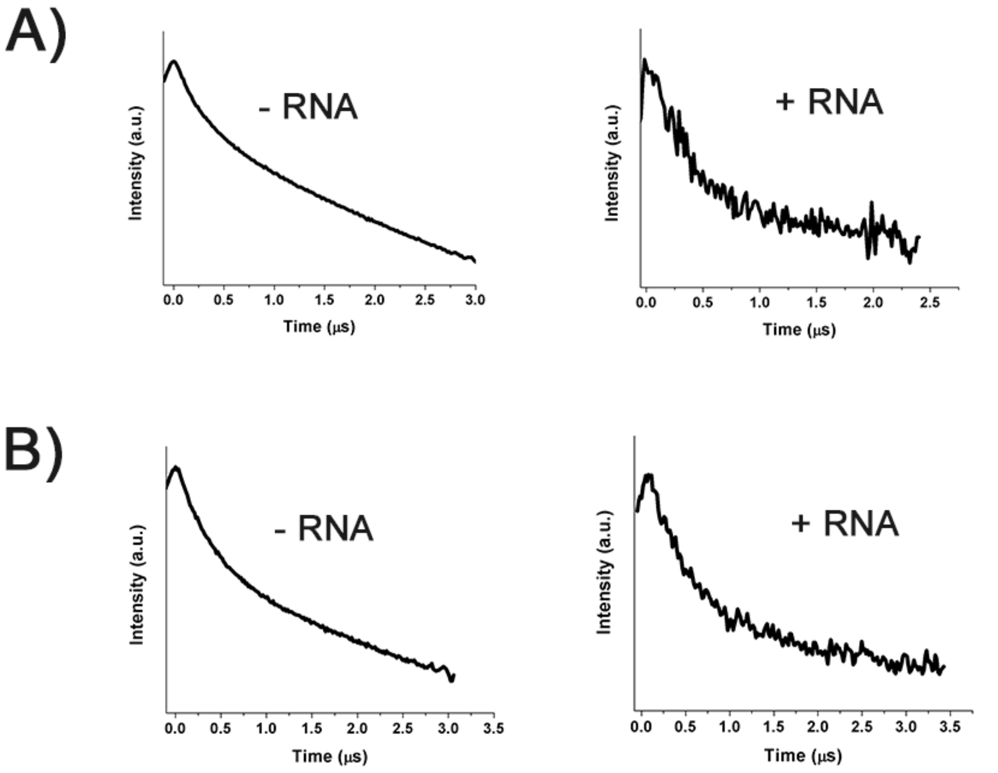

Supplementary Fig. S6: Raw Q-band DEER traces recorded for D258R1/A349R1 (A) and A349R1/S412R1 (B) labelled mutants of the $FUS^{162\text{-}422}$ construct with and without stem-loop RNA.
